# Ethical and legal issues in psychedelic harm reduction and integration therapy

**DOI:** 10.1186/s12954-021-00489-1

**Published:** 2021-04-07

**Authors:** Brian Pilecki, Jason B. Luoma, Geoff J. Bathje, Joseph Rhea, Vilmarie Fraguada Narloch

**Affiliations:** 1 Portland Psychotherapy Clinic, Research, & Training Center, 3700 N Williams Ave, Portland, OR 97227 USA; 2 Department of Counseling and Integrated Programs, Adler University, 17 N Dearborn, Chicago, IL 60602 USA; 3 Attorney, Palm Springs, CA USA; 4 Students for Sensible Drug Policy, 2370 Champlain St NW Ste #12, Washington DC, 20009 USA

**Keywords:** Psychedelics, Psychedelic-assisted therapy, Psychedelic integration, Harm reduction

## Abstract

Psychedelic-assisted therapy may represent an upcoming paradigm shift in the treatment of mental health problems as recent clinical trials have demonstrated strong evidence of their therapeutic benefits. While psychedelics are currently prohibited substances in most countries, the growing popularity of their therapeutic potential is leading many people to use psychedelics on their own rather than waiting for legal medical access. Therapists therefore have an ethical duty to meet this need by providing support for clients using psychedelics. However, incorporating psychedelics into traditional psychotherapy poses some risk given their prohibited status and many therapists are unsure of how they might practice in this area. This paper explicates such risks and describes ways in which therapists can mitigate them and strive to practice within legal and ethical boundaries. A harm reduction approach will be emphasized as a useful framework for conducting therapy around clients' use of psychedelics. It is argued that therapists can meet with clients before and after their own personal psychedelic experiences in order to help clients minimize risk and maximize benefit. Common clinical scenarios in this growing clinical area will also be discussed.

## Background information on psychedelic therapy, harm reduction, and integration

While psychedelic use has thrived for thousands of years in Indigenous cultures and for many decades in underground subcultures, psychedelics are increasingly being encountered in the mainstream in countries around the world, as evidenced by a surge of media attention on the therapeutic use of psychedelics, including popular books written on the topic [1]. The potential application of psychedelics in combination with therapy for the treatment of mental health difficulties has been studied in at least 10 published placebo-controlled, randomized trials to date, with early results appearing promising [2]. As a result of these and other events, many individuals suffering from mental health issues are now seeking to utilize psychedelics for healing, especially in cases where currently available psychotherapeutic or psychopharmacological treatments have failed. The resulting public demand for the therapeutic use of psychedelics is being felt in mental health services around the world. While there are no known studies documenting this increase in demand, one can look to the recent upsurge of psychedelic integration training programs, workshops, and referral networks as evidence of this increased public interest, as well as the high number volunteers for clinical trials on psychedelics.

Legal access to psychedelics for therapeutic purposes is not widespread largely because most psychedelics remain controlled substances in most countries. As a result, individuals seek underground (i.e., illegal) therapy, travel to countries where psychedelics are legally accessible, or use psychedelics on their own, with trusted friends, or with significant others. In any case, there is a growing need for education about psychedelics within the mental healthcare field so that clinicians can meet clients' current needs in this area. In this paper, we explore ways that therapists encountering clients who are interested in the therapeutic use of psychedelics can ethically and effectively meet this demand inside current legal and ethical regulatory contexts in the USA, such that client risk of harm is reduced and likelihood of positive outcomes is increased. While this paper will focus on a US context, many of these principles will generalize to practice in other countries.

Traditional or "classic" psychedelics are serotonergic agonists that include lysergic acid diethylamide (LSD), mescaline, dimethyltryptamine (DMT), and psilocybin [3]. Other substances with some of the same effects are often grouped together with classic psychedelics, including 3,4-methylenedioxymethamphetamine (MDMA) and ketamine. Because of this overlap in use and effect, the broader usage of the term "psychedelic" will be used throughout this paper to refer to substances that can result in profound shifts in states of consciousness. Many of these substances are naturally derived and have had a history of use in spiritual and healing contexts for thousands of years. Others are synthetic, but regardless of how they were produced, all have been used extensively for their spiritual, therapeutic, or healing effects.

Psychedelics are widely consumed throughout the world, with one recent survey estimating a prevalence of 32 million lifetime psychedelic users in the USA [4]. Many members of the public seek to use psychedelics for therapeutic purposes but are unsure of how to do so in a way that is physically, psychologically, and legally safe. In most countries, both classic and non-classic psychedelics remain controlled substances, though there are some exceptions, such as psilocybin's legal status in Jamaica and the Netherlands [5]. Some legal use occurs in the context of research trials, ketamine clinics, and religious groups using peyote and ayahuasca (a plant-based beverage containing DMT and other psychoactive substances). In addition, those with more money and privilege may choose to travel to countries where psychedelics are legal. The lack of a clear legal path toward the therapeutic use of psychedelics increases risks for people who are seeking to use psychedelics for this purpose.

Therapists have an ethical duty to try to reduce the risk of harm among clients who are interested in exploring or currently using psychedelics and a general duty to attempt to maximize the benefits of therapy; however, how therapists can best do this is unclear. In most countries, clinicians cannot yet prescribe psychedelics or serve as a guide during psychedelic experiences.^1^ However, with some legal and ethical considerations they can provide psychotherapy before and after a client has a psychedelic experience on their own. Many therapists have already been providing this service as evidenced by referral networks, such as Psychedelic. Support in the USA, that have been rapidly expanding over the last several years. The current paper is intended to serve as a resource for therapists working with clients who are seeking psychedelic experiences for personal benefit or therapeutic growth, though harm reduction is more broadly relevant to all personal use of any substance.

We argue that a harm reduction approach is ideally matched to this context. A harm reduction approach helps clients understand the potential risks and benefits of psychedelic use, contemplate alternative methods to reach desired goals, develop realistic expectations, and create intentions that help maximize therapeutic benefit of psychedelic use. A harm reduction approach acknowledges client autonomy and supports them in reducing risk and maximizing benefits from whatever life choices they make. As such, it also includes the possibility for psychotherapy after psychedelic experiences in which clinicians may meet with clients to help them process the experience, clarify gained wisdom or insights, provide support for challenges that may have occurred, and translate their experience into meaningful long-term change. In the current paper, we outline this approach, including legal and ethical considerations in working with people seeking psychedelic experiences. We begin with a background and history of psychedelic therapy. We then review research findings from past and recent trials and how psychedelic-assisted psychotherapy is conducted in most studies. Next, we discuss harm reduction broadly and how it can be applied to the context of psychedelics. We outline the most common risks for therapists engaging in this work and steps practitioners can take to mitigate risk. Finally, we discuss common decision points and reflection questions for clinicians considering involvement in this type of work.

## Background and history of psychedelic use and scientific research

Psychedelics have been used as tools for spiritual purposes, healing, and growth for thousands of years, likely as far back as prehistoric times [6, 7]. Psychoactive substances such as psilocybin found in mushrooms [8] and mescaline found in cacti [9] have played important roles in many cultures around the world. Western/European and scholarly work on psychedelics appears to have begun when mescaline was identified in the peyote cactus in the late nineteenth and early twentieth centuries [10]. The discovery of LSD in 1943 by Albert Hoffman in Switzerland further catalyzed interest in psychedelics by greatly increasing access among clinicians, scientists, and the public [11]. During the 1950s and 1960s, psychedelic research flourished as scientists attempted to understand more about the psychedelic experience and how it could potentially be used for therapeutic benefit in the treatment of mental illness (for a review, see Grinspoon and Bakalar [12]). While much of this early research took place in the USA, psychedelic research also took place in other Westernized countries including the Czech Republic [13] and Canada [14].

As clinical research was proliferating, psychedelics became more widely used in recreational settings in the USA and influenced mainstream culture, as evidenced in music and literature. However, there was a cultural backlash against psychedelics largely due to politics, misinformation, and fear. In 1970, the USA placed psychedelics in Schedule I of the Controlled Substances Act, deeming them to have no medical value, and this prohibition spread internationally which rapidly halted psychedelic research. Later that decade, MDMA, a non-classic psychedelic, was discovered and used as an adjunct to psychotherapy [15]. However, MDMA met a similar fate and was classified as a Schedule I drug in 1985 in the USA [16] and subsequently around the world one year later [17]. Despite their prohibited status, both classic and non-classic psychedelics continued to be used for therapeutic, exploratory, and recreational purposes in the "underground," a term referring to established communities, norms, and practices that were secretive in nature to protect individuals from potential legal consequences or stigma.

Research into classic psychedelics as treatment for mental health problems tapered off in the 1970s and remained dormant for several decades. However, advocates such as the Multidisciplinary Association for Psychedelic Studies (MAPS) persisted in efforts to obtain legal permission to conduct psychedelic research and there has been a recent resurgence of well-controlled clinical trials providing strong preliminary evidence for the use of some psychedelics for therapeutic purposes. A recent meta-analysis of nine rigorous placebo-controlled trials of psychedelic-assisted therapy was conducted and found a “very large” between-groups effect size of 1.21 (Hedges *g*) which is notably larger than average effect sizes for standard treatments using psychotherapy or psychopharmacological medications [2]. While previous decades of psychedelic research have focused on a wide array of psychological problems using many different psychedelic substances, modern research has demonstrated the strongest evidence for psychedelic-assisted therapy in the treatment of post-traumatic stress disorder (PTSD) [18], depression [19], end-of-life distress [20], addiction [21–23], and social anxiety in adults with autism [24]. While the evidence base continues to grow, it should be noted that many of these studies are preliminary trials with relatively small sample sizes (10–50 participants) and highly controlled inclusion and exclusion criteria. Furthermore, there are almost no studies investigating mechanisms or processes of change in psychedelic-assisted therapy. Clinicians practicing in this area should have an understanding of the current state of the evidence as part of ethical practice.

## The role of psychotherapy in psychedelic medicine

There is consensus on the need for psychotherapeutic support for people undergoing treatment using psychedelics [25, 26]. In clinical trials, this typically takes the form of a few preparation sessions, therapeutic support during dosing sessions, and a few integration sessions after each dosing session. Preparation sessions include the establishment of a therapeutic relationship, an exploration of participants' mental health issues, and discussion of participants’ intentions for dosing sessions [27]. Information is provided about the substance and its effects, potential risks and benefits, and strategies are identified for responding to difficult experiences that may show up during the dosing sessions.

Dosing sessions in trials typically last 6–8 h and usually have two therapists present. Clients listen to instrumental music, wear eyeshades, and are encouraged to focus on their internal experience [28]. Therapists are advised to adopt a "non-directive approach" that facilitates the "inner healing intelligence" of participants [29]. Therapists are also trained in how to effectively respond to difficult experiences a participant may have, including fear, confusion, uncomfortable somatic experiences, or attachment issues. Finally, therapists monitor safety and make sure the basic needs of participants, such as comfort or hydration, are met.

In clinical trials, integration sessions tend to be non-structured and focused on allowing participants to process and make meaning of memories, feelings, or ideas that were experienced during the dosing session [30, 31], including any challenging or confusing aspects. Integration may also help participants incorporate new understandings of their symptoms, changes in how their symptoms are experienced, or new insights into how to better manage symptoms [21]. The use of creative modalities such as drawing, painting, and writing are often encouraged. Physical movement via yoga or somatic therapies may be utilized, or participants may be encouraged to spend contemplative time in nature. Integration sessions also heavily focus on translating gained insights into meaningful, lasting change. Finally, integration sessions can help clients decide on next steps such as planning future psychedelic sessions, developing new personal practices (e.g., meditation, prayer, or exercise), or increasing social engagement.

One aspect of this model that should be emphasized is the high degree of care that experimenters take in curating the psychedelic experience. The physical environment is carefully arranged to maximize various important factors such as safety, comfort, and aesthetics. Participants are highly screened using selective exclusionary criteria such as a history of bipolar disorder or psychosis. Participants are supported by two professionals with developed trusting relationships. Participants are prepared with strategies for how to navigate challenges and therapists are trained to help implement those techniques. Participants are able to plan for sessions so as to optimize their state of mind entering the psychedelic experience. This is in sharp contrast to the way that psychedelics are often taken in less controlled or recreational settings, where experienced professionals are usually unavailable to provide support. For example, if an individual takes psychedelics at a concert or festival, this is often not carefully planned, there is likely little control over environmental variables such as weather, crowds, or even having a place to sit. There may be increased anxiety about engaging in an illegal activity and no trained individuals to assist in case of difficulty. Having a "bad trip" is not an uncommon result of such uncontrolled factors and can result in significant psychological distress or even trauma [32]. It seems probable that the rate of adverse events is greatly increased in these less controlled settings, compared to clinical trials where there have been very few adverse events. As just one example, of the 54 volunteers who received high doses of psilocybin in the Johns Hopkins studies, none demonstrated persisting problems related to their sessions and all were able to return to their normal daily activities [25]. In all, existing data and experience indicate that the preparation for psychedelic administration (i.e., set), support during administration (i.e., setting), and therapeutic follow-up (i.e., integration) are extremely important to avoid adverse events and increase the probability of beneficial effects.

## Harm reduction and psychedelics

Given the increased demand for psychedelic medicine and the importance of psychological support in producing good outcomes, many therapists have begun offering therapy services in this area. However, because the use of psychedelics in clinical practice, with the exception of ketamine, entails illegal activity, most therapists are hesitant to engage directly in providing components of psychedelic-assisted therapy. A harm reduction approach has long been established as an ethical and legal means for working with people who use substances and are not interested in or capable of complete abstinence [33]. We will now describe a harm reduction approach and outline its application to people seeking therapeutic support in using psychedelics for personal growth or healing purposes.

### History of harm reduction

Harm reduction approaches refer to a focus on reducing the negative consequences of drug use, rather than focusing on eliminating the use of the drug [34]. Harm reduction grew in response to limitations of abstinence-only approaches to drug use, the US war on drugs, and the acquired immunodeficiency syndrome (AIDS) epidemic where needle exchanges were observed to reduce risk among injection drug users [35]. While harm reduction can refer to a public health or social justice movement [36], it has been integrated in psychotherapy approaches for treating individuals engaging in risky behavior [37–39]. Harm reduction assumes that it is better to provide space for clients to be honest about their substance use with a therapist who is nonjudgmental and has their best interests in mind, rather than establishing a situation in which clients need to either terminate therapy or hide their use or lack of commitment to abstinence to avoid judgment or treatment rejection. In harm reduction approaches, therapists adopt a non-coercive stance and help clients identify the risks and benefits of their behaviors. Clients are treated with dignity and respect and are empowered to make their own choices. If abstinence is not a client goal, clients may instead be encouraged to alter the route of administration, use a safer substance, change other behaviors surrounding use, or reduce the frequency and intensity of substance use [40].

One common criticism of harm reduction is that by adopting a stance acknowledging that substance use can have both risks and benefits and providing a place for clients to openly discuss their drug use, therapists are condoning risky behavior and increasing the potential for harm to occur. However, the evidence suggests that harm reduction interventions have been effective in various areas [41] including preventing HIV in people who use drugs [42] needle exchange programs [43], opioid substitution therapy [44], and alcohol misuse in college students [45]. Further research has shown that harm reduction programs do not increase drug use and simultaneously increase treatment entry [46].

### Applying harm reduction to psychedelics

It is important to be clear that a harm reduction approach to psychedelic use does not permit therapists to legally attend or facilitate dosing sessions, something that is often called “guiding.” In the section below, we only have space to outline the basic principles and strategies involved in the application of harm reduction therapy in the context of psychedelics and refer readers to other sources [39, 47] for a more complete understanding of the harm reduction approach.

In contrast to the preparation work usually provided in clinical trials, harm reduction sessions before psychedelic use are oriented more toward helping clients make informed choices about psychedelic use and focus more heavily on safety and education. Clients who seek professional guidance in relation to psychedelics often have little experience or knowledge with these substances and are unsure whether psychedelic use is a good idea for them. In a harm reduction approach, the therapist does not advocate for or against the use of psychedelics, but instead focuses on the client’s goals and welfare and attempts to help the client determine for themselves what behaviors will lead them toward the life they desire. Consistent with this aim, the therapist often begins with helping the client clarify reasons for seeking an illicit psychedelic experience and may suggest alternative pathways for achieving desired goals, such as suggesting that a client seeking relief from depression first consider more established approaches such as psychotherapy, antidepressants, FDA approved psychedelic clinical trials, or ketamine treatment. From a harm reduction standpoint, these options would be presented to facilitate an informed choice.

Education about psychedelics, including their risks and benefits, is an important part of clients having informed consent as well as reducing risks associated with their use. In today's age, there is a large amount of information and misinformation on psychedelics that can be overwhelming to sort through. Clinicians can help in two ways. First, clinicians can provide resources, ask clients to do their own research, and provide space for clients to synthesize information they encounter. Clinicians can play a role in encouraging clients to critically evaluate information that they obtain on their own and help clients distinguish between fact and fiction. This may be especially important with psychedelics as informal information passed along by peers may be more trusted than information from healthcare resources. The situation may be similar to communities of people using performance-enhancing drugs, such as how growth hormone continues to be used by athletic communities despite strong evidence that it does not improve physical performance [48]. Second, clinicians can directly educate. The benefit of having clients do their own research is that clients may more clearly experience their therapists as objective if therapists do not provide information that could be perceived as approving or disapproving of psychedelic use. This may support client autonomy but may also be frustrating or confusing for clients who are seeking professional guidance and want direct information without needing to conduct their own research. Even if a therapist attempts to avoid appearing in favor of or against psychedelics, there still may be a need to provide information on risks that the client doesn’t discover on their own. For example, clients may think psychedelics are a “magic bullet,” unaware of the potential for challenging experiences or the emergence of avoided problems, memories, or emotions.

One common topic about which clients seek information is the potential interactions between psychedelics and medications they are currently taking. Unless the therapist is a prescriber, therapists should generally coach clients to bring such questions directly to their medical provider or assist clients in obtaining a psychedelic-friendly provider that would be willing to provide relevant information. Therapists working with clients considering withdrawing from medication should generally advise them to do so under the supervision of a prescriber. At minimum, clients can be helped to find online resources so that they can understand potential risks and benefits. While most psychedelics appear to have fewer unwanted side effects than many drugs [49], there are potential interactions between psychedelics and psychotropic medications. Some dangerous combinations are known such as the potentially lethal consequence of serotonin syndrome when using a serotoninergic substance such as Ayahuasca and certain antidepressants [50]. Clients should be aware that an absence of evidence does not guarantee safety, and that they may be taking on some risk if using psychedelics while on medications. Therefore, therapists are encouraged to network with local medication providers knowledgeable in psychedelics so that potential referrals can be made.

If clients decide to pursue psychedelic use, clinicians can be helpful in promoting safety by helping clients think through plans. Will they have support from someone they trust? Will they be in a safe, familiar environment where they won't need to drive? Will their physical needs be properly looked after, including diet and hydration? In addition, because psychedelics are prohibited substances, they are commonly purchased from sources that may be of questionable quality. While clinicians cannot facilitate access to psychedelics, they can encourage clients to be safe, including promoting the use of drug checking (also sometimes referred to as pill testing or reagent testing). Drug checking refers to the use of commercially available products that are legal in most countries and easily available for consumers to purchase in order to test the chemical makeup of various substances and identify whether what they purchased may be adulterated or actually another substance. However, clinicians should be aware of local laws since drug checking kits are considered paraphernalia in some jurisdictions, and their possession may be criminalized. While drug checking can help reduce risk, it may be subjective or imprecise and does not guarantee safety [51]. However, there is evidence to suggest that drug checking, when conducted in a laboratory by qualified staff (not using home-based testing kits), is helpful in reducing medical risks associated with taking drugs that may be adulterated or mixed with other dangerous chemicals [52].

Finally, harm reduction principles may be applied to helping clients who seek services from underground guides. Clients may be unaware that there is no regulatory oversight of underground guides or may fail to understand the risks of trusting another person while in a highly vulnerable state during psychedelic experiences. Clients can be informed about these potential risks and, if they choose to pursue obtaining an underground guide, clinicians can help clients assess the safety and trustworthiness of the guide. Clinicians can collaborate with clients to develop a set of questions to ask underground guides so that clients feel empowered to make a choice that is right for them. Clients may also benefit from encouragement to “trust their gut” if an underground guide does not feel safe and that they have the right to decline to proceed with a guide’s services at any point during the process, even if they have already committed time or money. Clients who experience harm from guides may also benefit from therapist assistance in determining whether or how to report a guide to legal or regulatory authorities as a means to prevent future harm to others.

Besides focusing on safety, a harm reduction approach may also serve to maximize the potential benefits of psychedelics. For decades, psychedelics have been used by individuals who value their beneficial effects and have integrated their use into a growth-oriented lifestyle. Using the dualistic model of passion, it is possible to view such use of psychedelics as a type of harmonious passion, or ongoing engagement with an activity that enhances life, rather than obsessive passion, which is a type of engagement with an activity that can interfere with other life domains [53]. This passion model offers an alternative to the abstinence-only or "all drugs are bad" approach that, instead of pathologizing the use of psychedelics, considers that their use may lead to positive emotions and psychological well-being. The passionate model has been applied to MDMA [54] and marijuana [55, 56] suggesting that some patterns of regular use may indeed be experienced as positive and are not necessarily associated with negative outcomes. A focus on benefit maximization in harm reduction therapy appears even more acceptable in the case of classic psychedelics given their well-documented low potential for dependence or physical harm [57]. In other words, because classic psychedelics are typically non-addictive and physically safe, the benefit-to-harm ratio is more likely to be weighted more toward the benefit side compared to some other mind-altering substances. Therefore, clinicians may be less inclined to focus on elucidating the potential harms of classic psychedelics and freer  to consider that use of these substances is potentially beneficial for clients. Harm reduction approaches allow clinicians to make space for clients to discuss positive consequences of the use of psychedelics and integrate them into their lives. Finally, harm reduction work may also entail psychedelic integration that is similar to therapy sessions in clinical trials which involve helping clients maximize benefit from their experiences.

### Risks associated with conducting psychedelic harm reduction and integration therapy

Many therapists who wish to offer psychedelic harm reduction and integration therapy (HRIT) are unsure of the potential level of risk involved and may therefore hesitate to provide this service. The following section will outline some of the most common types of risk associated with this emerging clinical area but is by no means an exhaustive account of all forms of risk that are possible. In addition, types and degrees of risk will differ depending on local regions, professional licensing boards, and dispositions of local law enforcement.

Perhaps the largest domain of risk relates to licensing boards. Even though a clinician may not engage in behavior that violates the law, a licensing board has greater latitude to assess and determine if a clinician is acting outside of the boundaries of acceptable professional practice. For example, guidelines in many US states include holding that licensed practitioners should not act in an unprofessional, unethical, or negligent manner. Due to the novelty of psychedelic therapy, less familiarity with harm reduction principles, and stigma against drug use, it is possible that any given licensing board may disapprove of therapists who are not explicitly trying to prevent people from using prohibited substances. Because licensing boards may receive complaints from clients, other clinicians, or general members of the public, there are multiple ways that they may become aware of a clinician’s actions. For example, a client's family could discover that the client has been meeting with a therapist for HRIT and perceive that the therapist has encouraged the client to use illicit drugs. Or a client may experience an adverse event during a psychedelic experience, such as physical injury or extreme psychological distress, and communicate this to another provider who knows that psychedelics have not been advocated against in therapy. Such a situation might trigger a report to be made, especially in states that require reporting of perceived unethical or unprofessional behavior by colleagues. In addition, a licensing board may consider it a violation to engage in intention setting or other strategies aimed at maximizing benefit as those might be perceived as encouraging clients to engage in illegal activities. Finally, some boards have guidelines that prohibit activities that may lead to negative perceptions of the profession by the public. For example, psychologists in the USA who are taking public positions on controversial issues are encouraged to consider potential negative consequences that may result from public perception of their profession [58]. As a parallel, the personal conduct of US medical physicians is also considered to be relevant to their professional role and reflective of the field in general [59].

To the authors’ knowledge, no licensing boards in the USA have taken disciplinary action against clinicians in relation to harm reduction therapy around psychedelics [33], but this does not mean that following a harm reduction approach will guarantee protection. Though ethical codes may differ between disciplines (e.g., psychology, counseling, social work), they all emphasize the importance of practicing within boundaries of competency. For example, a licensing board may want to know what education or training experiences have prepared the therapist to offer HRIT. Unfortunately, most licensing boards will not provide clear guidelines about practicing in this area. At the very least, complaints, even if without merit, may lead to stress and inconveniences, such as having to indicate that you have been previously investigated for unethical behavior when applying for malpractice insurance or insurance panels.

Another area of risk is criminal prosecution, which in the USA includes local, state, and federal levels. First Amendment protections for free speech extend to healthcare practitioners and discussion of information around drugs, such as when it was found that doctors could discuss the benefits of cannabis to their patients in California before cannabis was legalized [60]. This case found that discussion of marijuana would not constitute an anticipatory offense or accomplice liability, thus suggesting that therapists might be equally permitted to engage in discussion of psychedelics with their clients. However, if a therapist assists in the attainment of prohibited drugs or refers a client to an underground guide, this protection would no longer apply and could implicate one in racketeering, conspiracy to commit a crime, or aiding and abetting unlawful acts. Some therapists attempt to skirt laws by allowing clients to come to session under the influence of psychedelics that they have taken on their own and then conduct therapy with them during the experience. However, this would not be protected under the First Amendment and could be perceived as conspiracy in committing a crime or a violation of drug house laws which prohibit the provision of space to consume controlled drugs. In a conversation with a defense attorney in Oregon, a general rule pertaining to controlled substances was discussed, namely that “the more involvement you have and the more intrinsic your involvement is, the more risk you have of prosecution” (A. Margolis, oral communication, August 2020). It is generally not recommended to infer risk levels based on peer-group actions, which may be especially true in the context of the lack of clear guidelines about HRIT from licensing boards. In other words, just because a lot of peers are doing something, that behavior could still be sanctioned if reported to a licensing board or prosecuted if presented to legal authorities.

Another type of risk involves potential litigation of malpractice. If a client were harmed while using psychedelics, we can imagine three different grounds for civil suits that therapists may want to consider when making decisions about risk. First, the therapist could be sued for failing to protect the client from harm. In addition, lawsuits might be filed under the grounds that psychedelic HRIT is a new treatment that lacks sufficient scientific evidence. Finally, it could be argued that the therapist has violated standards of care by not taking a more conventional approach to treatment. Any of these grounds could also be a part of a licensing board decision.

Another type of risk involves that of professional reputation amongst peers and communities. This risk likely differs widely based on factors such as geographic location and type of workplace. This may be especially true in more conservative regions or traditional therapeutic contexts such as abstinence-focused substance use disorder treatment. If clinicians perceive that publicizing HRIT services might jeopardize their income or employment, it may limit the accessibility of this type of therapy for the public. In addition, some agencies might not support or permit this type of practice. Clinicians interested in providing psychedelic HRIT therapy are encouraged to think through these risk factors and consider other forms of risk that may be unique to their own personal circumstances.

## Steps to mitigate risk

While it is not possible to completely eliminate risk when practicing HRIT, it is important to think about steps that can be taken to mitigate such risk and increase protective factors. Even though harm reduction approaches for substance use disorder treatment are well-established and evidence-based, any area of practice involves its own degree and types of risk that must be managed. In relation to psychedelics, standards of ethical practice are not well established, licensing boards do not provide specific direction, and boundaries of criminal prosecution are not clear. However, there are some activities that clearly increase risk that one should be aware of.

A first and central way to avoid risk is to avoid facilitating access to psychedelics or prohibited substances in any way and avoid providing a space wherein psychedelics would be used. This includes recommending websites, such as those found on the dark web, to obtain prohibited substances or referring clients to individuals who sell psychedelics. Therapists should also refrain from communications that clients could see as suggesting they take psychedelics before a session and come to session under the influence. Although a clinician could claim this was not premeditated and they were only acting in the best interest of their client by not turning them away in a vulnerable state, it is possible that evidence suggesting this was planned or part of a pattern of behavior could implicate the clinician in violating drug house laws. In general, any activity that could lead a client to claim that the clinician directly assisted in their obtaining a prohibited drug or that the client purchased or used the drug because of the clinician’s involvement could contribute to legal risk.

Another way to avoid risk is to refrain from coordinating work with underground guides. Referring a client to an underground therapist is a clear and obvious form of knowingly facilitating access to prohibited substances. While from a clinical standpoint, it might be helpful for an underground guide to consult with a client's therapist before a psychedelic experience, this creates greater legal risk. In contrast, receiving referrals from an underground guide entails less legal risk as members of the public are free to refer to whatever practitioner they wish. However, consulting with underground guides on an ongoing basis through forming informal or formal arrangements to prepare clients for guiding or receive clients post-guiding entails greater risk as, again, this could be perceived as conspiracy in that the therapist has made themself part of someone else's drug deal by channeling or referring people to a drug source. These activities also entail risk of licensing board sanction. In general, any action that increases the chance a client claims their use of a prohibited drug was because of the involvement of a licensed practitioner might result in a licensing board determining that the clinician was an integral part of the illegal experience under the guise of providing professional services (thereby violating duty of care and acting in an unprofessional or unethical manner). Thus, preparation work may entail greater risk than integration work because it increases the chance a client could claim they only followed through with the drug experience because of interaction with the therapist.

Therapists can also reduce risk through carefully considering the language used on advertising, forms, and in documentation. First, it is important to be consistent in all written materials that the clinician does not facilitate access to controlled or prohibited drugs. It is advisable to avoid certain terms such as “preparation” or “guide” to increase clarity and reduce the probability that someone may misperceive HRIT therapy as involving the administration of psychedelic substances. It may be helpful to have a separate informed consent document that unambiguously describes what HRIT work is and is not. This may include outlining procedures that a clinician will implement if a client comes to session under the influence of psychedelics, including contacting the client’s emergency contact and arranging for safe transport home. Clearly specifying boundaries of practice in all verbal communication can be helpful as well, as some clients may think that your website guidelines may just be formalities that are then overlooked once they meet you. Therefore, having direct conversations upfront when screening potential clients can be helpful in dispelling any confusion about services you provide.

Another important risk reduction strategy that is part of ethical practice is to expand the boundaries of one’s competency by obtaining adequate training and access to consultation resources. Currently, there is an expansion of programs from which to receive training or education on conducting harm reduction work around psychedelics, but due to lack of consensus on minimal training standards, it can be difficult to decide what training is necessary. While this will likely change over the next several years, clinicians may be frustrated by the lack of clear pathways to increase education. More general training in harm reduction approaches for substance use is also helpful. Finally, clinicians should consider supervision and consultation with other professionals that are more experienced in HRIT or who share similar interests.

As previously mentioned, risk varies by state and profession. Therefore, it is advisable for clinicians to investigate their local contexts before offering HRIT therapy. This may include learning more about one’s licensing board, laws related to controlled drugs, and community attitudes about psychedelics. Are there other providers in the area who are doing this work? What would some colleagues think if you advertised in this area? Criminal risk is most likely to come from local law enforcement, so it is advisable to meet with a local attorney specializing in criminal defense to understand the local context. Thinking through all of these factors can be helpful for clinicians in making a decision about whether or not to engage in this domain of practice.

Finally, it is recommended that clinicians become familiar with the empirical support for psychedelic-assisted therapy, including both strengths and limitations. Knowing this body of evidence is helpful in talking to clients about psychedelics in a way that avoids making claims that are exaggerated or inflated, especially in the light of current media portrayals of psychedelics as a “cure-all” or “breakthrough treatment” that may create unrealistic expectations of benefit. It is therefore helpful to know the science and be able to speak from an informed, balanced, and evidence-based perspective. For example, psychedelics have only shown efficacy in treating a small number of conditions and there are still many unknowns about why psychedelics may be beneficial or how they work. Speaking to clients from this perspective demonstrates sound ethical practice.

### Know the law and know thyself: common decision points encountered by therapists

Below we outline common situations that often occur in HRIT therapy relating to psychedelics. We also discuss factors to help clinicians in decision-making. It is recommended that readers consider these situations and anticipate how they might respond based on their own unique circumstances. In general, we recommend an approach of "Know the Law and Know Thyself." This means taking time to engage in self-inquiry to reflect on your aims for clinical practice, local laws and regulations, and the degree of risk that you might be willing to take on. For example, a 45-year-old therapist with student debt and a large family may make a different personal choice than a financially secure semi-retired professional with no dependents.

Many clients who present for HRIT are seeking or have found underground guides to provide them with psychedelic substances and be present with them during their experience. Ideally, a guide and therapist would work together if psychedelics were legal. While this collaboration between guide and therapist might be clinically optimal and tempting to carry out, it increases risk for any therapist who is attempting to practice within the boundaries of legal and professional guidelines. It is possible that clients who are unaware of these factors may request this coordination of care or have difficulty understanding why a clinician may decline to speak with their guide. A conservative approach for asserting professional boundaries might include telling clients that you are unable to have any contact with their underground guides because of potential legal sanctions.

HRIT also includes providing information about the risks and benefits of psychedelics. Therapists will need to commonly refer clients to educational resources and are encouraged to spend some time developing their own resource list so that they are comfortable with the information that they are referring clients to. It is also recommended that therapists provide a wide range of informational sources. For example, a client may want to know what dosage of psilocybin mushrooms to ingest and may be directed to some online resources to gather information in various forms such as guidelines from the clinical trials, "trip reports,” or suggestions from experienced users. When referring clients to resources such as websites, books, or podcasts, it is important to preface that these materials are for educational purposes and are not being provided as treatment recommendations. Many websites or organizations such as erowid.org, rollsafe.org, dancesafe.org, or tripsafe.org attempt to provide balanced and accurate information about the potential effects and side effects of many types of psychedelic substances. Though clinicians should be at least somewhat familiar with any resource they share with clients, it is not necessary to personally ensure that all information on any given website is completely accurate.

Clients who have financial resources may ask a clinician's opinion about traveling to other countries where psychedelic retreats are legal. Indeed, psychedelic tourism is expanding rapidly in countries throughout the world [5]. While this option may circumvent legal restrictions, traveling to international retreat centers carries unique risks. First, organizations that offer retreats may not comply with professional standards that exist in a client's country of origin. Retreat centers can vary widely in terms of quality and organization, and often exist as tourism entities that are not subject to healthcare regulation within the countries that they operate in. Second, some retreat centers may have negligible procedures for screening or preparing participants and therefore may include individuals who are not adequately prepared or stable enough for psychedelic experiences. Moreover, there have been reported instances of sexual abuse perpetrated by shamans or guides in psychedelic contexts [61]. For these reasons, it is recommended that clients considering traveling to other countries familiarize themselves with guidelines for reducing such risks, such as traveling with trusted companions, conducting thorough research, and understanding norms for psychedelic ceremonies (e.g., that nudity or sexual touch is not part of traditional ayahuasca ceremonies). Therefore, it is recommended that clinicians exercise caution in making specific referrals to retreat centers that could be interpreted as endorsements.

Finally, clients sometimes ask about clinicians' prior experience with psychedelics. This may be more common in relation to psychedelics than other drugs, perhaps because people who have used psychedelics are often aware that these altered states are difficult to understand for people who have not experienced them. Clinicians may be concerned that disclosing past use may involve admitting engagement in illegal activity. Clinicians may feel that questions regarding their own use of psychedelics infringes on their personal life and may therefore be reluctant to answer. Guidelines for self-disclosure vary based upon theoretical orientation and clinician background and, ultimately, one’s response to such a question is a personal choice and may differ from client to client or the clinical context. One option is to provide a vague answer that affirms one's prior experience without going into detail such as stating, "I have experience with altered states of consciousness." Clinicians offering HRIT are encouraged to think through how they might respond to such inevitable questions.

## Diversity, equity, and inclusion in harm reduction and integration therapy

Ethical practice needs to consider the larger sociopolitical context surrounding psychedelics, particularly with issues related to diversity, equity, and inclusion (DEI), as well as social justice. Psychedelic-assisted therapy and the current psychedelic renaissance is embedded within a White-dominant medical framework [62]. There is a significant lack of diversity within the field of psychedelic researchers, with Indigenous people and people of color underrepresented both as researchers, therapists, and participants in studies [63]. Psychedelic science has been criticized for its lack of cultural humility, or the acknowledgment that we are bound by limitations of our social backgrounds and often unaware of our own privilege [64]. This lack of diversity stands in stark contrast to the fact that psychedelics have been part of the spiritual practices and cultures of Indigenous people throughout the world and have historically been frequently condemned by Western cultures. As a result, the "discovery" of psychedelic-assisted therapy by Western medicine has been criticized as another example of colonialism or cultural appropriation that repeats a history of oppression [65].

We also wish to note that this paper highlights the role of psychotherapy in reducing risk and maximizing benefit in relation to psychedelics, but we do not mean to imply that Western forms of therapy have a monopoly on the psychedelic experience. Rather, we believe that many of the essential elements of effective support for psychedelic use are contained in Indigenous, spiritual, or religious cultures that have been using psychedelics long before psychotherapy existed. In sum, psychotherapy models, which have often been informed by these older traditions, are one way to gain benefit from psychedelics, but not the only one.

When psychedelic-assisted therapy becomes legal, it will likely be very expensive with estimates of a course of MDMA treatment in the USA to be around $15,000, a cost that may not be covered by health insurance [66]. As a result, it is likely that psychedelic medicine will be inaccessible to individuals of lower socioeconomic status (SES), putting barriers to access in marginalized populations. Consequently, it is likely that there will remain an active underground use of psychedelic medicines for individuals who may not be able to afford the legal versions.

Fortunately, the growth of psychedelic medicine, especially in the US current sociopolitical context of increased awareness of racial/ethnic and gender inequality, affords an opportunity to diverge from existing systems of oppression and take measures to promote values associated with DEI. Clinicians engaging in HRIT should familiarize themselves with the lack of diversity in psychedelic research and understand limits of generalizing results to people not well-represented in the research. Clinicians need to acknowledge the power and privilege associated with roles in dominant groups such as in educated White men. Clinicians are encouraged to do their own personal work around cultural humility and understand how their own history, experience, and biases may impact their clinical work. In addition, clinicians should adapt their therapy approach to clients who identify as people of color or who have other marginalized identities and remember that the notion of therapy itself is often perceived as a "White space" by many individuals. For example, in adapting MDMA-assisted psychotherapy to be more culturally informed, clinicians are encouraged to spend more time in initial screening to counteract potential mistrust toward the medical system that is common in groups that have formerly been mistreated by medical professionals, as well as to formally assess racial trauma and institutionalized oppression [67]. Finally, clinicians who belong to groups that hold greater power and privilege are at less legal and regulatory risk when conducting HRIT and should consider that those with less power may be justifiably concerned about their safety in considering open practice in this area. Thus, clinicians who hold power and privilege should consider ways in which they can support clinicians with less privilege in doing this work and to help dismantle stigma and bias about the potential therapeutic benefits of psychedelics. Finally, it’s important to keep in mind that clients from less advantaged backgrounds will likely experience higher risk of criminal prosecution should they face legal consequences for their involvement with psychedelics. Good resources for learning more about the importance of DEI in psychedelics can be found in Williams and Labate [68] and George et al. [62].

## Personal inquiry and conclusion

We end with emphasizing the importance of personal reflection by clinicians involved in this area of work. We refer readers to a list of reflection questions to help clinicians think through their potential interest in psychedelic medicine that can be found in the “Appendix”. Questions include prompts to consider reasons for conducting work related to psychedelics, clarifying values related to this newer clinical area, assessing competency, pursuing additional training and education, and taking next steps.

Psychedelic-assisted therapy has been touted as a potential paradigm shift in the treatment of mental health problems [69]. Access to legal psychedelic medicine will also likely increase personal or "underground" use as more people become aware of psychedelics. Therapists have an obligation to become better educated and prepared to incorporate psychedelic experiences into their practice so that individuals who choose to use psychedelics can be better supported when seeking therapy. We hope that this paper will contribute to a growing collection of ethical and professional guidelines for therapists interested in HRIT. Future directions might include establishing a code of ethics for HRIT, similar to the code of ethics that was developed for MDMA-assisted psychotherapy [70]. Other future directions include the need for efforts to reduce the stigma associated with psychedelics so that people who use psychedelics can be more open about their use and therapists can better understand how to serve the needs of this population. Finally, we encourage therapists to consider advocacy work in roles or positions that can help advance policy based on harm reduction principles and ideas outlined in this paper.

## Data Availability

Not applicable.

## Appendix: Personal inquiry for clinicians considering psychedelic Harm Reduction and Integration Therapy (HRIT)

Pilecki, B., Luoma, J.B., Bathje, G.J., Rhea, J., & Narloch, V. F. (in press). Ethical and Legal Issues in Psychedelic Harm Reduction and Integration Therapy. *Harm Reduction Journal.*

The following section contains suggestions for clinicians who are considering becoming more involved in harm reduction and integration therapy for the use of psychedelics. These reflection questions are meant to prompt self-inquiry and to help clarify potential next steps in increasing involvement with HRIT.

*What are my reasons for doing work that relates to psychedelics?* Clinicians may want to clarify their own set of values that draws them to this work and make sure that it is an appropriate fit. Though psychedelics at first might seem like an interesting and attractive area to pursue, it is like any other clinical specialty that includes both pros and cons and is better suited for different people. It is helpful to identify what is meaningful to you about practicing in this area, as well as what aspects of it might be difficult or aversive. Taking some time to think through what a caseload of individuals engaged in psychedelics might entail will be helpful in readying oneself to do this work.

*Am I comfortable with psychedelic content?* The psychedelic experience can involve aspects of spirituality and mysticism, supernatural occurrences, or experiences with entities or beings. This type of content might be off-putting to some clinicians. It might be helpful to reflect on any recent clinical encounters you have had involving clients who have brought up similar topics and how comfortable you were in discussing them. In addition, psychedelic experiences can include intensely felt emotions and expressions of affect. Clinicians are encouraged to assess their level of comfort in bringing the types of experiences individuals with psychedelics frequently have into the therapy encounter and whether that is of interest to them.

*What is my level of competency in working with psychedelics and harm reduction? What might I do to develop competency?* Currently, there are limited professional arenas for obtaining education and training in this area, though this is likely to change as psychedelic-assisted therapy becomes more widely disseminated. The Multidisciplinary Association for Psychedelic Science (MAPS) conducts training for individuals delivering MDMA in clinical trials. In the USA, other educational centers providing training and workshops in areas of harm reduction and psychotherapy include Fluence and The Center for Optimal Living. There are also several programs that provide training in psychedelic-assisted therapy including the California Institute of Integral Studies (CIIS), the Medicinal Mindfulness Psychedelic Sitters School, and Salt City Psychedelic Therapy & Research (SCPTR). Other options include volunteering for a harm reduction organization such as the Zendo Project which provides psychological support for participants of music festivals and concerts who are having difficult psychological experiences. Clinicians are encouraged to think about potential deficits in their knowledge base related to psychedelics and peruse research articles or media in these areas. For example, perhaps there is a particular psychedelic substance that you don’t know much about or really want to better understand what is meant by the term “mystical experience.” Or, perhaps you are less familiar with harm reduction in general and would benefit from general training in more traditional applications of this treatment approach. Another option for learning more about psychedelics is to read “trip reports” online, which are personal accounts by users of psychedelics detailing their experiences.

*What level of risk am I comfortable taking on?* HRIT involves some level of legal and regulatory risk. We suggest clinicians reflect on the level of risk that they are willing to assume if conducting HRIT and steps they can take to mitigate such risks. Clinicians are encouraged to consider their own unique situations, local laws and guidelines, and then determine what activities they might be willing or not willing to engage in. For example, some clinicians may feel that talking about psychedelics is too uncomfortable for them and that they therefore might not be effective in working with clients in this area. Or, some clinicians may feel that there is too great of a professional or legal risk in conducting HRIT given their workplace situation or local law enforcement context.

*What is my personal level of experience?* Many clinicians who are new to psychedelics are curious as to whether or not they need to have their own prior experiences with psychedelics. This is a delicate manner, as we cannot recommend doing anything that would be illegal. However, there are advantages to having experience with non-ordinary states of consciousness given their unique phenomenology. In the MAPS training program for MDMA-assisted therapy, many of the first cohorts of therapists received their own MDMA experience as part of the training. In the 1950s and 1960s, it was also the norm that individuals studying psychiatry or psychedelic substances would take psychedelics themselves, although this norm for psychiatry is not currently in favor. Therefore, therapists without personal experience are encouraged to find legal ways to gain experience with non-ordinary states of consciousness such as by using methods like holotropic breathwork, mindfulness practice, spiritual retreats, or sensory deprivation tanks.

*What is my privilege and how safe is it for me to practice HRIT?* Depending on where you live, practicing HRIT may be considered controversial by colleagues and peers. For example, areas with conservative political attitudes or active religious communities may have stronger stigmatizing beliefs against psychedelics. In addition, one's unique identities may confer greater risk. It is possible that therapists who identify as women, low SES, BIPOC, LGBTQ+, or gender non-conforming may bear increased risk. Therefore, it is understandable that such therapists may be less comfortable in practicing HRIT. It is hopeful that as more professionals speak out and work toward counteracting stigma, HRIT will become a more widely accepted clinical area. Professionals who benefit from greater privilege are encouraged to think about the role that they would like to occupy in such efforts.

*What is my level of support?* If you are considering practice in HRIT, you might want to consider the degree of support you have from clinicians who are conducting this type of therapy. You may want to consider joining or forming a peer consultation group of other clinicians engaged in HRIT. Or, you may consider paid consultation from established experts in the field or joining a professional listserv that allows for the discussion of clinical issues.

*Am I interested in Policy Change?* Therapists with training and background in harm reduction can play a pivotal role in shaping and advancing public policy related to psychedelics and other drugs. There is a need for unbiased voices, free of stigma or misinformation, in conversations about policies that impact people who use psychedelics. You may consider taking on roles or positions that allow you to make such contributions.

## Abbreviations

AIDS: Acquired immunodeficiency syndrome (AIDS)
BIPOC: Black, Indigenous, and people of color
CIIS: California Institute of Integral Studies
DEI: Diversity, equity, and inclusion
DMT: Dimethyltryptamine
HRIT: Harm Reduction and Integration Therapy
LGBTQ+: Lesbian, gay, bi, queer
LSD: Lysergic acid diethylamide
MAPS: Multidisciplinary Association for Psychedelic Studies
MDMA: 3,4-Methylenedioxymethamphetamine
PTSD: Post-traumatic stress disorder
SES: Socioeconomic status

## Footnotes

1 One exception to this in the USA is ketamine treatment which is commonly prescribed in a non-psychedelic medical context, but is sometimes used in a context similar to psychedelic-assisted therapy. While ketamine is not a traditional psychedelic, it can have psychedelic properties depending on dose, set, and setting, and exists as a current legal alternative to psilocybin or MDMA-assisted therapy which are currently in Phase III clinical trials and not yet available to the public.
